# Apparent effective Text4Hope online mental health support for male subscribers during the COVID-19 pandemic- Naturalistic randomized controlled trial

**DOI:** 10.1192/j.eurpsy.2023.1821

**Published:** 2023-07-19

**Authors:** R. Shalaby, B. Agyapong, W. Vuong, V. Agyapong

**Affiliations:** 1 Department of Psychiatry, University of Alberta; 2 Addiction and Mental Health, Alberta Health Services, Edmonton, Canada

## Abstract

**Introduction:**

Mental illness is not uncommon among males. While the estimates of the males who are dissatisfied with their lives, die by suicide, become alcohol-dependent and drug users are high, low numbers seek mental health support.

**Objectives:**

In this study, we aimed to assess Text4Hope, a texting mental health support service, provided to people in Alberta during the COVID-19 pandemic, and examine its effectiveness among male subscribers.

**Methods:**

In a naturalistic randomized controlled trial design, a comparison was run between two populations of Text4Hope male subscribers; an intervention group (IG, Text4Hope subscribers who received once-daily supportive text messages for 6 weeks) and a control group (CG, Text4Hope subscribers who joined the program in the same time frame but were yet to receive text messages). The severity and the prevalence of likely stress, anxiety, and depression were examined between the two groups, using the Perceived Stress Scale (PSS-10), the Generalized Anxiety Disorder 7-item (GAD-7), and the Patient Health Questionnaire-9 (PHQ-9), and defined the Composite Mental Health (CMH) score as the sum of these three. T-test, Chi-squared association, and binary logistic regression analyses were applied.

**Results:**

There were 286 male subscribers to Text4Hope. The majority were above 40 years, white, employed, had postsecondary education, were in a relationship, and owned a home. Mean scores of PSS-10, GAD-7, and PHQ-9 scales and the CMH were significantly higher for the CG compared to the IG, 11.4, 28.8, 25.8, and 18.7%, respectively. Similarly, there was a statistically significant lower prevalence in IG, compared to the CG, on likely MDD (58.15 vs. 37.4%) and likely GAD (50 vs. 30.8%), with a small effect size. The IG was a significant predictor for lower odds of both likely MDD and likely GAD while controlling for sociodemographic characteristics.

**Conclusions:**

Text4Hope is an effective tool for mental health support for male subscribers, during the COVID-19 pandemic. Compared to the subscribers who didn’t receive the service, those who received it were in a better mental health condition. Further efforts are still needed to encourage males to participate in such online services that can provide adequate support, particularly during crisis time.

**Disclosure of Interest:**

None Declared

